# Impact of Intra-Retinal Fluids on Changes in Retinal Ganglion Cell and Nerve Fiber Layers in Neovascular AMD under Anti-VEGF Therapy

**DOI:** 10.3390/jcm13175318

**Published:** 2024-09-08

**Authors:** Yaser Abu Dail, Berthold Seitz, Haris Sideroudi, Alaa Din Abdin

**Affiliations:** Department of Ophthalmology, Saarland University Medical Center, 66421 Homburg, Germany; berthold.seitz@uks.eu (B.S.); hsideroudi@gmail.com (H.S.); alaadin.abdin@uks.eu (A.D.A.)

**Keywords:** intraretinal fluids, retinal ganglion cell layer, retinal nerve fiber layer, anti-VEGF intravitreal injection, age related macular degeneration

## Abstract

**Purpose**: To investigate the influence of intraretinal fluid (IRF) on change in retinal nerve fiber layer (RNFL) and retinal ganglion cell layer (RGCL) and thickness in patients with naive neovascular AMD under anti-VEGF treatment. **Design**: post hoc analysis. **Methods**: 97 eyes of 83 patients on continuous therapy with intravitreal anti-vascular endothelial growth factors (anti-VEGF) and a follow-up of 24 months were included. RGCL and RNFL thickness in the perifoveal (-O), parafoveal (PF), and nasal areas and number of injections (IVI) were recorded before the first IVI as well as 1 and 2 years after initiating treatment and compared longitudinally and between groups with and without IRF. **Results**: The group with IRF at baseline had a higher RNFL thickness at baseline and showed a significant reduction in RNFL-PF between baseline and first and second follow-ups (*p* < 0.001) but not between first and second follow-ups. The group without IRF showed no significant reduction in RNFL over time. The presence of IRF was not associated with a reduction in RNFL-O or RNFL-nasal. RGCL thickness decreased significantly in both groups with and without IRF after 2 years. Number of IVIs showed no significant correlation to RNFL or RGCL after stratification for the presence of IRF. **Conclusions**: The presence of IRF has a significant influence on RNFL thickness at baseline as well as on its changes over time during anti-VEGF therapy. The preoperative presence of IRF should be considered when comparing changes in RNFL thickness after IVI.

## 1. Introduction

Intravitreal injection (IVI) of anti-vascular endothelial growth factors (anti-VEGF) is the standard therapy for neovascular age-related macular degeneration (nAMD) [1,2]. Their efficacy in improving functional and anatomical outcomes in nAMD patients has been demonstrated in several clinical trials [3,4,5,6,7]. However, as they frequently require long-term use, their long-term effects on the retinal nerve fibers layer (RNFL) and retinal ganglion cell layer (RGCL) are of increasing interest [8].

Several studies have investigated the effect of anti-VEGF treatment on RGCL [9,10,11,12,13,14,15,16,17] and on RNFL thickness [9,11,13,14,15,17,18,19]. However, the results were inconsistent [8].

One possible explanation for this inconsistency is that the therapeutic reduction in intraretinal fluid (IRF) with anti-VEGF treatment varies depending on the prevalence of such fluids at baseline in different study populations.

Indeed, studies that examined only lesion-free areas (areas without IRF and retinal pigment epithelium atrophy (RPE-atrophy)) showed no significant reduction in RGCL and RNFL thickness in these areas [9,15], suggesting that various nAMD manifestations play an important role in the observed changes in RGCL and RNFL thickness.

Our previous study also showed a significant negative correlation between the reduction in RGCL thickness and the presence of RPE-atrophy, further emphasizing the role of nAMD manifestations in the observed reduction in RGCL and RNFL thickness [8].

Understanding the impact of such factors (IRF, RPE-atrophy) and taking them into account when selecting patients for comparative trials, will allow for better comparisons between the growing number of anti-VEGF drugs [20,21,22].

Hence, our intention was to further understand the impact of IRF on the changes in RGCL and RNFL thickness with anti-VEGF therapy. Since we found no difference between ranibizumab, aflibercept and bevacizumab regarding their impact on RGCL and RNFL in our previous study, nor in terms of rates of presence or resolution of IRF between them [8], we combined these three medication groups into a total group to increase the statistical power and performed a post hoc analysis.

The purpose of this study was to investigate the influence of the presence of IRF on the changes in RGCL and RNFL thickness in patients with naive neovascular AMD under anti-VEGF treatment.

## 2. Methods

Details of the design and methods of the original study has been published previously [8]. The study was conducted in accordance with the Declaration of Helsinki and approved by the Ethics Committee of the Medical Association of Saarland, Germany (Nr. 123/20) in 2020. Because of the retrospective design of the study, written informed consent was waived.

We included patients with nAMD (macular neovascularization types 1 and 2) who were continuously treated with either ranibizumab (Lucentis, Novartis Pharma GmbH, Nuernberg, Germany), bevacizumab (Avastin, Roche Holding AG, Basel, Switzerland), or aflibercept (Eylea, Bayer Pharma AG, Berlin, Germany) and had a follow-up period of 2 years with a spectral-domain optical coherence tomography (Spectralis SD-OCT; Heidelberg Engineering, Heidelberg, Germany) in our intravitreal injection (IVI) center [23] with the auto-rescan mode on. Patients excluded were those with glaucoma, an image quality-reducing cataract, ocular hypertension, any intraocular pressure (IOP) measurement > 24 mmHg before or during the follow-up period, uveitis, diabetic macular edema, moderate or severe non-proliferative as well as proliferative diabetic retinopathy, myopic macular neovascularization, retinal artery and/or vein occlusion or a history of photodynamic therapy, ocular trauma, pars-plana vitrectomy or rtPA lysis. The pervious diseases and treatments may confound the changes in thickness of RNFL and RGCL.

Demographic data, the number of IVIs which has been administered at 1- and 2-year follow-ups and the type of administered anti-VEGF medication were collected. Best corrected visual acuity (BCVA), measured using Snellen charts, intraocular pressure before the injection, and the presence of IRF were recorded at the start point and at 1- and 2-year follow-ups.

Since the decimal visual acuity chart is not the standard and difficult to use for statistical analysis, we converted the BCVA values to logMAR units to match the current standard in the literature.

A total of 97 eyes from 83 patients were included in this post hoc analysis. The analysis investigated the development of RGCL and RNFL thickness parafoveally (-PF) and perifoveally (-O). To investigate the impact of IRF on the RGCL and RNFL thickness, the entire group was separated into two groups, with IRF (N = 51) and without IRF (N = 46) present at baseline. The correlation between the number of IVIs on one side and RGCL and RNFL thickness on the other side was also examined. To further investigate the influence of IRF on the changes in RGCL and RNFL thickness under anti-VEGF therapy, the IRF group was divided into two subgroups: a group with resolved IRF after 2 years (resolved IRF, N = 41) and a group with persistent IRF after 2 years (persistent IRF, N = 10). Subsequently, a sub-analysis was performed to compare the changes in the previous subgroups.

An SD-OCT was used to acquire the retinal scans. Macular volumetric retinal scans included 19 parallel B-scans 240 µm apart with a pattern size of 20° × 15° and were carried out using the automatic real-time repeat function. Baseline OCT was conducted directly before administering the first IVI. Scans 1 year and 2 years after initial anti-VEGF treatment were acquired using the auto-rescan follow-up feature. B-scans were averaged eight times. The minimal image quality required for B-scans was 15 dB, as indicated by the manufacturer [24]. RGCL and RNFL thickness were initially acquired using the auto-segmentation function of the Heidelberg Eye Explorer (version 1.10.0.0). The B-scans were then reviewed by an experienced retinologist for segmentation errors in the internal limiting membrane (ILM), RGCL, RNFL and Bruch’s membrane (BM) and adjusted manually if required [25]. Mean RGCL and RNFL thicknesses of the inner (r: 0.5 to 1.5 mm) and outer rings (r: 1.5 to 3 mm) were calculated based on the implemented Early Treatment Diabetic Retinopathy Study Grid (ETDRS). The parafoveal area (-PF) was considered as the area of the inner ring of the ETDRS grid. The perifoveal area (-O) was considered as the area of the outer ring of the ETDRS grid.

SPSS version 25 was used for statistical analysis. Continuous data were expressed as means and standard errors of the mean, and categorical variables were expressed as percentages. Categorical variables were compared using the chi-square test. For continuous variables, since they were normally distributed, the paired samples *t*-test, one-way and two-way ANOVA were used. For non-parametric variables, the Wilcoxon and Kruskal–Wallis tests were applied. For time series analysis, repeated measures were used for normally distributed variables and Friedman’s test for non-parametric variables. Pearson and Spearman correlations were applied to assess the association between parametric and non-parametric variables, respectively. In addition, a general linear model with estimated marginal means including the indicated variable, compared groups and three timepoints: Baseline, 1-year, and 2-year follow-ups with Bonferroni adjustment for multiple comparisons was implemented where applicable. Patients with comorbidities which might influence RGCL and RNFL were already excluded from the study. Hence, further adjustment for these potential confounding factors was not required. A *p*-value of less than 0.05 was regarded as statistically significant. The Bonferroni adjusted *p*-value was used for multiple comparisons.

## 3. Results

Baseline characteristics and changes in RGCL and RNFL thickness in the total group are summarized in Table 1 and Table 2, respectively.

### 3.1. RGCL Thickness

In the group with IRF at baseline, RGCL-PF thickness significantly decreased at 1-and 2-year follow-ups compared to baseline and between 1- and 2-year follow-ups [baseline–1 year = 2.3 µm (4.8%), baseline–2 year = 3.0 µm (6.4%), 1–2 year = 0.74 µm (1.6%)] (Table 3), while the reduction in RGCL-PF thickness in the group without IRF at baseline was significant only after 2 years [baseline–2 year = 1.1 µm (2.4%)] (Table 3). The difference between the two groups was not significant at all time points (Table 3).

**Figure 1:**
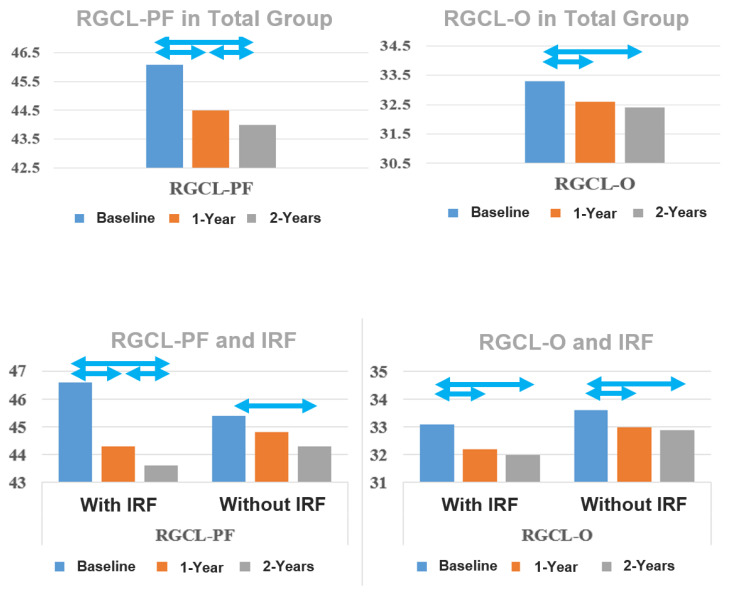
Development of the thickness of the retinal ganglion cell layer (RGCL) parafoveal (PF) and in the outer ring of the ETDRS grid (O) in the overall group and in the groups with and without subretinal fluid (SRF). Statistically significant changes (*p* < 0.05) are marked with double arrows.

In both groups with and without IRF at baseline, RGCL-O thickness decreased significantly at 1- and 2-year follow-ups compared to baseline, with no significant difference between these groups at all time points. There was no statistically significant difference between 1- and 2-year follow-ups (Table 3 and Figure 1).

### 3.2. RNFL Thickness Development

In the group with IRF at baseline, RNFL decreased significantly at 1- and 2-year follow-ups compared to baseline, without a significant difference between the 1- and 2-year follow-ups [baseline–1 year = 1.49 µm (5.2%), baseline–2 year = 1.62 µm (5.7%), 1–2 year = 0.13 µm (0.5%)], while in the group without IRF, RNFL-PF showed no significant difference over time [baseline–1 year = 0.63 µm (2.5%), baseline–2 year = 0.42 µm (1.7%), 1–2 year = −0.21 µm (−0.8%)] (Table 4).

In the group with IRF, RNFL-O decreased significantly at 1-year follow-up compared to baseline (Table 4). However, there was no significant reduction in RNFL-O between baseline and 2-year follow-up, nor between 1- and 2-year follow-ups (Table 4). The group without IRF at baseline showed no statistically significant changes over time (Table 4).

**Figure 2:**
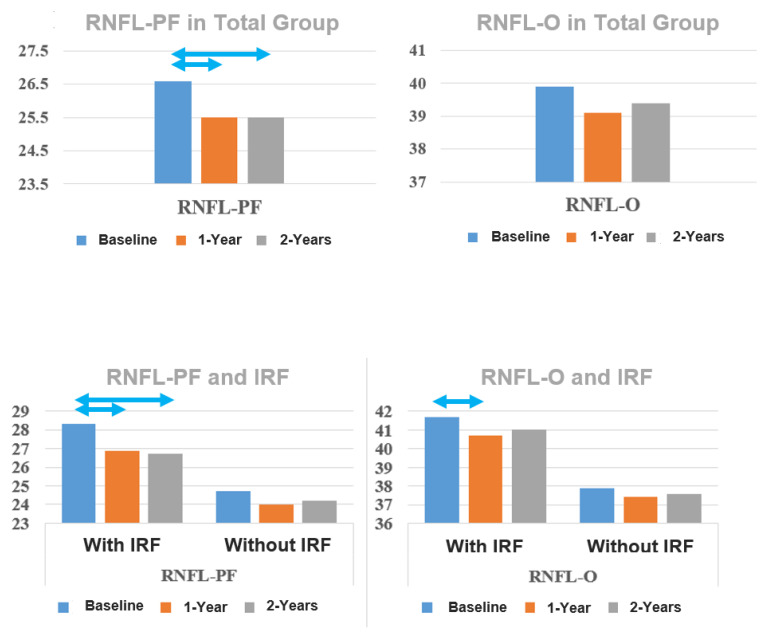
Development of the thickness of the retinal nerve fiber layer (RNFL), both parafoveal (PF) and in the outer ring of the ETDRS grid (O), in the overall group, and in the groups with and without intraretinal fluid (IRF). Statistically significant changes (*p* < 0.05) are marked with double arrows.

Interestingly, RNFL thickness in the outer ring nasally (RNFL-nasal) remained relatively stable at 1 and 2 years in the total group and in the groups with and without IRF (Table 2 and Table 4, respectively) (Figure 2).

### 3.3. BCVA

BCVA was significantly worse in the IRF group compared to the group without IRF at all time points (Table 5).

### 3.4. Intraretinal Fluids

The percentage of eyes with IRF decreased significantly in the total group from 53% at baseline to 21% and 11% at 1- and 2-year follow-ups, respectively (*p* < 0.001) (Table 2). The difference between 1- and 2-year follow-ups was not statistically significant (*p* = 0.29) (Table 2).

### 3.5. Number of IVIs

The number of IVIs at 1- and 2-year follow-ups was significantly lower in the group with IRF at baseline compared to the group without IRF (Table 1).

The number of IVIs showed a significant, negative, and weak correlation only to RNFL-PF thickness at 2-year follow-up (R = −0.222, *p* = 0.029) (Table 6). However, in a sub-analysis of the correlation between RNFL-PF at 2-year follow-up and IVIs in the two groups with and without IRF, the correlation was weaker and not significant in either of the groups (group without IRF: R = −0.151, *p* = 0.315, group with IRF: R = −0.078, *p* = 0.587).

### 3.6. Results of the Sub-Analysis of the IRF Group

The resolved IRF group showed changes in RGCL and RNFL thickness similar to the total IRF group. On the other hand, the persistent IRF group showed no statistically significant changes in RGCL and RNFL thickness over the 2-year follow-up period (Table 7).

## 4. Discussion

In this post hoc analysis, we combined the three medication groups from our previous study [8] to increase statistical power and examined the possible influence of IRF as well as the number of IVIs on the changes in RGCL and RNFL thickness.

**RGCL thickness:** Several studies explored the effect of anti-VEGF treatment on RGCL thickness [9,10,11,12,13,14,15,16,17].

The results of these studies varied depending on the macular area examined (parafoveal, perifoveal) and the inclusion or exclusion of lesion-free areas of nAMD. Studies investigating RGCL-PF with the inclusion of lesion areas [12,13,14,16], including our study, all found a statistically significant reduction in thickness over periods between 1 and 2 years of follow-up. In contrast, studies that examined RGCL-O with the inclusion of lesion areas [10,11,14,16] disagreed. Beck et al. [10], Lee et al. [11], and Aşikgarip [14] found a statistically significant reduction in RGCL-O thickness over a mean period of 44, 19, and 12 months, respectively. However, in the study of Abdolrahimzadeh et al. [16], the reduction in RGCL-O in treatment group was not statistically significant compared to the reduction in the control group after 24 months of follow-up. In our study, although both RGCL-PF and RGCL-O showed a significant reduction in thickness at 1-year and 2-year follow-up compared with baseline, the difference between the 1-year and 2-year follow-ups was significant only in RGCL-PF but not in RGCL-O, indicating a different pattern of thickness development and possibly a difference in etiology behind the two patterns of reduction. On the other hand, the two studies that investigated lesion-free areas [9,15] found no significant decrease in the inner retina thickness (including RNFL and RGCL) over a 1-year follow-up period.

One possible explanation for the previous discrepancies in results is that local processes, such as the presence of IRF and RPE atrophy [9,15], and their uneven distribution in the paravofeal and perifoveal areas, play an important role in the observed decrease in RGCL thickness. Additionally, the perifoveal area has more than three times the surface of the parafoveal area, which makes it less prone to detectable change due to local processes such as RPE-atrophy and IRF. This is likely reflected in the unanimous reduction in RGCL-PF thickness in studies that included areas with nAMD lesions, the lack of reduction in RGCL thickness in studies that examined only lesion-free areas, and in the discrepancies seen in the results concerning changes in the RGCL-O area. In addition, the sub-analysis of the IRF group showed no significant reduction in RGCL-PF nor RGCL-O thickness in the persistent IRF group. This is in contrast to the results in the resolved IRF group, in which RGCL-PF and RGCL-O thickness at the 1-year and 2-year follow-up appointments were significantly reduced compared to baseline. This emphasizes the influence of changes in IRF on the observed changes in RGCL thickness. The local, time-dependent role of RPE-atrophy RGCL loss is reflected in the negative, significant correlation between RPE-atrophy and RGCL-PF thickness but not RGCL-O thickness at 2 years follow-up in our previous study [8]. RPE-atrophy is caused in part by oxidative stress and the resulting inflammation in AMD patients. On the other hand, Beck et al. [10] found a negative significant correlation between RPE-atrophy and RGCL-O thickness, but after a longer mean follow-up time of 44 months.

Another observation in many of the previous studies [11,12,13,14,16] and in our study is that most of the RGCL thinning occurred between baseline and first follow-up. In the studies by Kim et al., 2020 and Abdolrahimzadeh et al. [12,16], as well as in our study, the first decrease was even statistically significant (Table 8). This reduction is better explained by a rapid decrease in IRF than by a slower degenerative process caused by RPE-atrophy or anti-VEGF therapy.

Age-dependent RGCL thinning has been reported in the literature [26,27]. However, Beck et al. [10], Aşikgarip et al. [14], and Abdolrahimzadeh et al. [16] found significant thinning in RGCL compared to baseline and to a control group of contralateral eyes, which indicates the presence of RGCL thinning under anti-VEGF therapy independent of the aging process.

The influence of intraocular pressure (IOP) spikes on RGCL and RNFL thickness has also been suggested in the literature and could also play a role in the observed thickness reduction [8,28,29].

**RNFL thickness:** There are also numerous studies that have investigated the effect of anti-VEGF treatment on RNFL thickness. These studies also differed in terms of areas studied, the follow-up time, the medication, and the presence of a control group.

When looking only at the results of treatment (study) groups, the results of studies are contradictory, regardless of the areas investigated (peripapillary or macular). Studies that examined the peripapillary area [30,31,32,33,34,35,36,37] were combined in 2 meta-analyses [18,19]. The meta-analysis of Shin et al. reported conflicting results regarding the correlation between intravitreal anti-VEGF treatment and RNFL thickness reduction. The more recent meta-analysis by de Vries et al. in 2019 reported a significant reduction in RNFL thickness of 2–3 µm over an average follow-up period of 23 months. Valverde-Megias et al. [38] also found a significant reduction in RNFL thickness in the study group over a 96-month period. The results of treatment groups in studies that explored the local changes in RNFL thickness in the macula with anti-VEGF therapy [9,10,11,13,14,15,17] are also conflicting. Inan et al. [13], Makri et al. [9] Demir et al. [15], Kim et al. [17], Beck et al. [10] and our study in general (RNFL-O, -nasal, and -PF without IRF), found no significant change in RNFL over time under anti-VEGF therapy. On the other hand, Lee et al. [11] and Aşikgarip et al. [14] found a significant RNFL thickness reduction with intravitreal anti-VEGF treatment.

Interestingly, when examining studies with a control group and a comparable baseline RNFL thickness between treatment and study groups, a sub analysis of Shin et al. [19] found no significant difference between study and control groups. In the study by Valverde-Megías et al. [38], the non-significance between study and control groups persisted even over an 8-year follow-up period. These results make a deleterious effect of anti-VEGF therapy on RNFL an unlikely cause of the observed decrease in RNFL thickness. Instead, some authors [34,38] discussed three explanations for the symmetric thinning of RNFL under anti-VEGF therapy: age-related RNFL loss [38,39,40,41,42], nonexudative AMD in the fellow eye [34], and/or contralateral effect of anti-VEGF due to systemic circulation. Contralateral thinning due to the systemic effect of anti-VEGF was justifiably considered unlikely by Valverde-Megías et al. [38] because the symmetry between control and treatment eyes was maintained over very long follow-up periods [10,38]. Although an influence of nonexudative AMD in the control eye is possible, symmetry was preserved when a healthy comparison group [35,37] was used. Hence, age-related thinning probably plays the main role in the observed RNFL loss over time.

Another important factor that our study highlights is the presence of IRF at baseline; the group with IRF showed a significant RNFL thickness reduction in the perifoveal area, where the effect of local factors like IRF is most apparent as discussed above, whereas the group without IRF showed no statistically significant reduction in RNFL-PF over 2 years of follow-up. Variation in IRF prevalence at baseline and therapeutic reduction in IRF under anti-VEGF therapy provide a good explanation for the discrepancy in the results of studies examining the macular area. Another indirect hint for the importance of IRF in the etiology of RNFL thinning is that both Makri et al. [9] and Demir et al. [15], who examined lesion-free retinal areas, found no reduction in RNFL and inner retina thickness under anti-VEGF therapy. In our study, we also found no significant decrease in the RNFL-O and RNFL-nasal areas, where the influence of IRF is usually less pronounced. A third indicator of the role of IRF is that the major reduction in RNFL thickness in the studies of Entezari et al. [43], Lee et al. [11], Aşikgarip et al. [14], Martinez-de-la-Casa et al. [31], and Inan et al. [13] happened in the first three months after treatment (Table 9). This is likely due to a rapid response to anti-VEGF therapy, which leads to a decrease in fluid leakage from macular neovascularization, resulting in a reduction in IRF in the retinal layers, including RGCL and RNFL, and thus a rapid reduction in their thickness. In addition, the sub-analysis of the IRF group showed no significant change in RNFL-PF thickness during the follow-up period in the persistent IRF group, in contrast to the resolved IRF group, in which RNFL-PF thickness was significantly reduced at the 1-year and 2-year follow-ups compared to baseline. This also underscores the influence of changes in IRF on the changes in RNFL-PF thickness over time. 

**Number of IVIs:** Several studies [10,11,16], including our study, investigated the association between RGCL thickness and number of IVIs and found no significant correlation. However, the results of studies investigating the correlation between RNFL and number of IVIs [10,11,34,35,37,44] were conflicting. Regardless of these results, the main problem with the number of IVIs as a parameter in real life studies is its dependence on the activity of nAMD [44]. The higher the activity and severity of the nAMD, the higher the number of IVIs required to control the disease. Therefore, the correlations between the number of IVIs and the thickness of the retinal layers in real-life studies are inherently confounded by the activity and severity of the disease. This is reflected in our results, as only RNFL-PF thickness showed a significant correlation with the number of IVIs. No such correlation was found between the number of IVIs and RNFL-nasal or RNFL-O thickness. However, a reduction in RNFL thickness due to a side effect of anti-VEGF IVIs would be expected to lead to a general reduction in RNFL thickness, not only in the PF area. A further analysis we performed, in which we stratified the presence of IRF at baseline, showed that the correlation between number of IVIs and RNFL-PF thickness was no longer significant in either group with or without IRF. This provides a convenient example of how a manifestation of nAMD (IRF) can confound a correlation with the number of IVIs.

**BCVA:** The presence of IRF at baseline in our study indicated a worse visual prognosis. These results are consistent with the results found by Waldstein et al. [45] in their post hoc analysis of VIEW trials. One possible explanation is that IRF may cause or correlate with permanent damage to the retinal layers, including RGCL and RNFL. Such damage could also play a role in reducing the thickness of the RGCL and RNFL areas exposed to IRF.

Based on the previous findings, we conclude that the thinning of RGCL and RNFL under anti-VEGF therapy is a multifactor process in which age-related loss, variations in IRF at baseline, and its therapeutic reduction under anti-VEGF therapy [38,46], RPE-atrophy, IOP spikes, and nAMD itself may play important roles. The process appears to involve a rapid reduction in thickness, likely due to the reduction in IRF, followed by a slower reduction due to age, RPE-atrophy, the effects of nAMD and, questionably, anti-VEGFs and IOP spikes. Although RNFL loss due to intravitreal anti-VEGF therapy cannot be excluded, it appears to be clinically insignificant. Further studies with control groups and the simultaneous consideration of the IVI and the presence of IRF and RPE-atrophy are needed to determine the presence and the span of the effect of intravitreal anti-VEGF therapy on RGCs.

While the influence of IRF on RNFL-PF was clear in the study, it was less obvious for the RGCL-PF. We assume that this is because RGCs are organized parallel to the measurement axis of layer thickness, whereas RNFs are perpendicular to it. Therefore, the intercellular fluids separating and distancing cells and fibers will cause a more detectable change in RNFL than in RGCL. However, further studies with better statistical power are needed to support these findings.

The strength of this study lies in the strict inclusion criteria. However, the study is limited due to it being a post hoc analysis as well as the retrospective design, and the fact that it was a mono-center study [8].

Further studies with a better statistical power and adjusting for age, number of injections, IRF, and RPE-atrophy presence, location and evolution over time are required to substantiate and quantify the influence of these parameters on RGCL an RNFL thickness over longer time periods.

## 5. Conclusions

The observed change in RGCL and RNFL thickness over time is possibly a multifactorial phenomenon with a fast early phase reduction due to the therapeutic decrease in IRF and a more gradual late phase decrease due to aging, downstream degeneration of RGCL and RNFL resulting from RPE-atrophy and/or as a side effect of anti-VEGF medications. The presence of IRF should be considered a confounding factor when examining changes in the thickness of RNFL.

## Figures and Tables

**Table 1:** Demographic and ocular characteristics.

	Total Group N = 97	IRF ^b^ N = 51	No-IRF ^b^ N = 46	*p*-Value ^c^
Age ^a^ (yrs) Mean ± SD ^b^	78.6 ± 7.0	79.2 ± 6.4	77.8 ± 7.7	0.326 ^d^
Gender % Male:Female	56:44	49:51	52:48	0.684 ^e^
Lens status % Pseudophakic:Phakic	58:42	75:25	48:52	**0.012** ^**e,f**^
Number of injections–12 months Mean ± SD	7.4 ± 1.5	7.0 ± 1.8	8.2 ± 1.8	**0.002** ^**d,f**^
Number of injections–24 months Mean ± SD	12.7 ± 3.6	12.2 ± 3.4	14.3 ± 4.3	**0.013** ^**d,f**^

^a^ Age at date of first intravitreal injection; ^b^ SD: standard deviation, IRF: group with intraretinal fluids at baseline, No-IRF: group without intraretinal fluids at baseline; ^c^ *p*-value for comparison between IRF and No-IRF groups; ^d^ *t*-test for equality of means; ^e^ chi-square test; ^f^ **bold** *p*-value indicates statistical significance (*p* < 0.05).

**Table 2:** Functional and morphological outcomes of the total group at baseline, 1-year, and 2-year follow-ups.

	Total Group N = 97			*p*-Value ^a^		
	Baseline	1-Year Follow-Up	2-Year Follow-Up	Baseline–1-Year	Baseline–2-Year	1-Year– 2-Year
RGCL-PF (µm) Mean ± SE	46.1 ± 0.6	44.5 ± 0.6	44.0 ± 0.6	**<0.001 ^b^**	**<0.001**	**0.012**
RGCL-O (µm) Mean ± SE	33.3 ± 0.4	32.6 ± 0.4	32.4 ± 0.4	**<0.001**	**<0.001**	0.180
RNFL-PF (µm) Mean ± SE	26.6 ± 0.4	25.5 ± 0.4	25.5 ± 0.4	**<0.001**	**0.001**	1.000
RNFL-O (µm) Mean ± SE	39.9 ± 0.7	39.1 ± 0.7	39.4 ± 0.7	0.249	0.299	0.193
RNFL-Nasal (µm) Mean ± SE	55.3 ± 1.1	54.8 ± 1.1	54.7 ± 1.1	0.249	0.299	0.193
BCVA logMAR Mean ± SE	0.47 ± 0.03	0.37 ± 0.03	0.40 ± 0.03	**0.001**	0.053	0.449
Percentage of IRF	53%	21%	11%	**<0.001**	**<0.001**	0.290

^a^ General linear model with estimated marginal means including the indicated variable, total group and three timepoints: baseline, 1-year, and 2-year follow-ups with Bonferroni adjustment for multiple comparisons. **^b^** **Bold** *p*-value indicates statistical significance (*p* < 0.05). Abbreviations: RGCL: retinal ganglion cell layer, RNFL: retinal nerve fibers layer, -PF: inner ring ETDRS grid (parafoveal), -O: outer ring of ETDRS ring (perifoveal), BCVA: best corrected visual acuity, SE: standard error, IRF: intraretinal fluid.

**Table 3:** Comparison of retinal ganglion cell layer thickness between eyes with and without intraretinal fluids at baseline at three time points: Baseline, 1-year, and 2-year follow-ups.

	Intraretinal Fluids		
	Yes N = 51	No N = 46	*p*-Value ^a^
RGCL-PF ^b^ (um) Mean ± SE ^b^			
Baseline	46.6 ± 0.8	45.4 ± 0.9	0.317
1-year follow-up	44.3 ± 0.8	44.8 ± 0.9	0.746
2-year follow-up	43.6 ± 0.9	44.3 ± 0.9	0.582
*p*-value ^a^			
Baseline–1-year	**<0.001** ^**c**^	0.243	
Baseline–2-year	**<0.001**	**0.013**	
1-year–2-year	**0.026**	0.454	
RGCL-O ^b^ (um) Mean ± SE			
Baseline	33.1 ± 0.5	33.6 ± 0.5	0.544
1-year follow-up	32.2 ± 0.5	33.0 ± 0.6	0.367
2-year follow-up	32.0 ± 0.6	32.9 ± 0.6	0.253
*p*-value ^a^			
Baseline–1-year	**<0.001**	**0.002**	
Baseline–2-year	**<0.001**	**0.004**	
1-year–2-year	0.104	1.000	

^a^ General linear model with estimated marginal means including the indicated variable, IRF and three time points: baseline, 1-year, and 2-year follow-ups with Bonferroni adjustment for multiple comparisons. ^b^ Abbreviations—RGCL: retinal ganglion cell layer, -O: outer ring of ETDRS ring (perifoveal), -PF: inner ring ETDRS grid (parafoveal), SE: standard error. **^c^ Bold** *p*-value indicates statistical significance (*p* < 0.05).

**Table 4:** Comparison of retinal nerve fiber layer thickness between eyes with and without intraretinal fluids at baseline at three time points: baseline, 1-year, and 2-year follow-ups.

	Intraretinal Fluids		
	Yes N = 51	No N = 46	*p*-Value ^a^
RNFL-PF (um) Mean ± SE			
Baseline	28.3 ± 0.6	24.7 ± 0.6	**<0.001** ^**b**^
1-year follow-up	26.9 ±0.5	24.0 ± 0.5	**<0.001**
2-year follow-up	26.7 ± 0.6	24.2 ± 0.6	**0.003**
*p*-value ^a^			
Baseline–1-year	**<0.001**	0.304	
Baseline–2-year	**<0.001**	0.882	
1-year–2-year	1.000	1.000	
RNFL-O (um) Mean ± SE			
Baseline	41.7 ± 0.9	37.9 ± 1.0	**0.006**
1-year follow-up	40.7 ± 0.9	37.4 ± 0.9	**0.012**
2-year follow-up	41.0 ± 0.9	37.6 ± 1.0	**0.012**
*p*-value ^a^			
Baseline–1-year	**0.013**	0.495	
Baseline–2-year	0.261	1.000	
1-year–2-year	0.868	1.000	
RNFL-Nasal (um) Mean ± SE			
Baseline	57.7 ± 1.5	52.7 ± 1.6	**0.027**
1-year follow-up	56.7 ± 1.5	52.7 ± 1.6	0.070
2-year follow-up	57.0 ± 1.6	52.1 ± 1.6	**0.035**
*p*-value ^a^			
Baseline–1-year	0.088	1.000	
Baseline–2-year	0.659	1.000	
1-year–2-year	1.000	0.576	

^a^ General linear model with estimated marginal means including the indicated variable, IRF and three time points: Baseline, 1-year and 2-year follow-ups with Bonferroni adjustment for multiple comparisons. Abbreviations: RNFL: retinal nerve fibers layer, -PF: inner ring ETDRS grid (parafoveal), -O: outer ring of ETDRS ring (perifoveal), SE: standard error. **^b^ Bold** *p*-value indicates statistical significance (*p* < 0.05)

**Table 5:** Comparison of best-corrected visual acuity between eyes with and without intraretinal fluids at baseline at time points: Baseline, 1-year, and 2-year follow-ups.

	Intraretinal Fluids		
	Yes N = 51	No N = 46	*p*-Value ^a^
BCVA logMAR Mean ± SE	N = 44	N= 42	
Baseline	0.54 ± 0.04	0.41 ± 0.04	**0.041** ^**b**^
1-year follow-up	0.45 ± 0.04	0.30 ± 0.04	**0.009**
2-year follow-up	0.47 ± 0.04	0.33 ± 0.04	**0.014**
*p*-value ^a^			
Baseline–1-year	0.067	**0.012**	
Baseline–2-year	0.441	0.181	
1-year–2-year	1.000	0.751	

^a^ General linear model with estimated marginal means, including the indicated variable, IRF and three time points: Baseline, 1-year, and 2-year follow-ups with Bonferroni adjustment for multiple comparisons. Abbreviations—BCVA: best-corrected visual acuity, SE: standard error. **^b^ Bold** *p*-value indicates statistical significance (*p* < 0.05).

**Table 6:** Correlation between number of injections and existence of RPE-atrophy at 24 months and ganglion cell layer thickness and retinal nerve fiber layer thickness at 24 months.

	RGCL-PF	RGCL-O	RNFL-PF	RNFL-O	RNFL-Nasal
Number of injections−24 Months R (*p*-value ^a^)	0.110 (0.284)	0.179 (0.079)	**−0.222** **(0.029)** ^**b**^	0.020 (0.846)	0.043 (0.675)

^a^ Spearman’s rho test. Abbreviations: RGCL: Retinal ganglion cell layer, RNFL: Retinal nerve fibers layer, -O: Outer ring of ETDRS ring (perifoveal), -PF: Inner ring ETDRS grid (parafoveal), R = Correlation coefficient. **^b^ Bold** *p*-value indicates statistical significance (*p* < 0.05).

**Table 7:** Comparison of retinal ganglion cell layer and nerve fiber layer thickness between eyes with resolved intraretinal fluids and persistent intraretinal fluids at 2-year follow-up at three time points: Baseline, 1-year, and 2-year follow-ups.

	Intraretinal Fluids		
	Resolved N = 41	Persistent N = 10	*p*-Value ^a^
RGCL-PF ^b^ (um) Mean ± SD ^b^			
Baseline	46.3 ± 5.7	47.6 ± 4.5	0451
1-year follow-up	43.8 ± 5.9	46.7 ± 3.8	0.070
2-year follow-up	42.8 ± 6.2	47.0 ± 5.3	**0.048**
*p*-value ^a^			
Baseline–1-year	**<0.001** ^ **c**^	0.559	
Baseline–2-year	**<0.001**	1.000	
1-year–2-year	**0.05**	1.000	
RGCL-O ^b^ (um) Mean ± SD			
Baseline	33.2 ± 3.8	32.8 ± 2.6	0.686
1-year follow-up	32.3 ± 3.7	32.1 ± 2.9	0.869
2-year follow-up	31.7 ± 3.9	32.9 ± 3.0	0.324
*p*-value ^a^			
Baseline–1-year	**<0.001**	0.295	
Baseline–2-year	**<0.001**	1.000	
1-year–2-year	**<0.001**	0.179	
RNFL-PF ^b^ (um) Mean ± SD			
Baseline	27.9 ± 4.0	29.9 ± 5.2	0.298
1-year follow-up	26.4 ± 3.4	28.6 ± 4.9	0.212
2-year follow-up	26.0 ± 3.6	29.5 ± 5.7	0.093
*p*-value ^a^			
Baseline–1-year	**0.014**	0.376	
Baseline–2-year	**0.002**	1.000	
1-year–2-year	0.559	1.000	
RNFL-O ^b^ (um) Mean ± SD			
Baseline	40.8 ± 6.5	45.0 ± 8.7	0.190
1-year follow-up	39.7 ± 6.1	44.3 ± 7.7	0.108
2-year follow-up	39.9 ± 6.2	45.3 ± 8.7	0.088
*p*-value ^a^			
Baseline–1-year	0.082	1.000	
Baseline–2-year	0.287	1.000	
1-year–2-year	1.000	1.000	
RNFL-Nasal (um) Mean ± SD			
Baseline	56.2 ± 9.7	63.4 ± 18.7	0.279
1-year follow-up	55.1 ± 9.6	63.0 ± 15.9	0.164
2-year follow-up	55.0 ± 9.9	65.0 ± 17.2	0.109
*p*-value ^a^			
Baseline–1-year	0.083	1.000	
Baseline–2-year	0.140	1.000	
1-year–2-year	1.000	0.545	

^a^ General linear model with estimated marginal means including the indicated variable, IRF and three time points: Baseline, 1-year and 2-year follow-ups with Bonferroni adjustment for multiple comparisons. ^b^ Abbreviations: RGCL: retinal ganglion cell layer, RNFL: retinal nerve fiber layer, -PF: inner ring ETDRS grid (parafoveal), -O: outer ring of ETDRS ring (perifoveal), -Nasal: the nasal area of the outer ring of ETDRS ring, SD: standard deviation. **^c^** **Bold** *p*-value indicates statistical significance (*p* < 0.05)

**Table 8:** Retinal ganglion cell layer thickness change over time in different studies.

	Baseline	First Control Thickness Change (%) (Months)	Last Control Thickness Change (%) (Months)	*p*-Value ^a^	*p*-Value ^b^
Lee et al., 2020 [11] (n = 52)	56.6 ± 10.7	53.0 ± 11.1 (−6%) (3 mo)	52.4 ± 10.9 (−7%) (19 mo)	0.098	**0.048**
Kim et al., 2020 [12] (n = 96)	70.5 ± 14.1	66.0 ± 13.9 (−6%) (3 mo)	62.6 ± 16.3 (−11%) (24 mo)	**0.004** ^**c**^	**<0.001**
Inan et al., 2019 [13] (n = 37)	44.5 ± 12.6	42.1 ± 12.7 (−5%) (3 mo)	39.6 ± 10.6 (−11%) (12 mo)		**0.005**
Aşikgarip et al., 2021 [14] (n = 36)	48.1 ± 7.1	46.1 ± 6.7 (−4%) (3 mo)	43.8 ± 6.1 (−9%) (12 mo)	0.223	**0.041**
Abdolrahimzadeh et al., 2019 [16] (n = 48)	49.4 ± 5.9	−2.1 ± 3.7 (−4%) (12 mo)	−3.0 ± 2.6 (−6%) (24 mo)	**0.01**	**<0.001**

^a^ *p*-Value of change between baseline and first control. ^b^ *p*-Value of change between baseline and last control. ^c^ **Bold** *p*-value indicates statistical significance.

**Table 9:** Retinal nerve fiber layer thickness change over time in different studies.

	Baseline	First Control Thickness Change (%) (Months)	First Control Thickness Change (%) (Months)	*p*-Value ^a^	*p*-Value ^b^
Lee et al., 2020 [11] (n = 52)	41.6 ± 14.4	35.7 ± 14.3 (−14%) (3 mo)	35.6 ± 13.6 (−14%) (19 mo)	**0.039** ^**c**^	**0.044**
Martinez-de-la-Casa et al., 2012 [31] (n = 49)	105.7 ± 12.2	101.4 ± 10.4 (−4%) (3 mo)	100.2 ± 11.0 (−5%) (12 mo)	**<0.001**	**<0.001**
Inan et al., 2019 [13] (n = 39)	29.3 ± 12.1	26.1 ± 8.9 (−11%) (3 mo)	26.9 ± 10.1 (−8%) (12 mo)	**-**	0.432
Aşikgarip et al., 2021 [14] (n = 36)	21.1 ± 2.7	20.7 ± 2.5 (−2%) (3 mo)	19.8 ± 2.4 (−6%) (12 mo)	0.515	**0.037**
Entezari et al., 2014 [43] (n = 18)	89 ± 21	82 ± 15 (−8%) (3 mo)	87 ± 23 (−2%) (6 mo)	**0.021**	0.356

^a^ *p*-Value of change between baseline and first control. ^b^ *p*-Value of change between baseline and last control. ^c^ **Bold** *p*-Value indicates statistical significance.

## Data Availability

The data that support the findings of this study are available on request from the corresponding author. The data are not publicly available due to privacy or ethical restrictions.

## References

[1] Mitchell P., Liew G., Gopinath B., Wong T.Y. (2018). Age-related macular degeneration. Lancet.

[2] Browning D.J., Kaiser P.K., Rosenfeld P.J., Stewart M.W. (2012). Aflibercept for age-related macular degeneration: A game-changer or quiet addition?. Am. J. Ophthalmol..

[3] Nguyen C.L., Oh L.J., Wong E., Wei J., Chilov M. (2018). Anti-vascular endothelial growth factor for neovascular age-related macular degeneration: A meta-analysis of randomized controlled trials. BMC Ophthalmol..

[4] Solomon S.D., Lindsley K., Vedula S.S., Krzystolik M.G., Hawkins B.S. (2019). Anti-vascular endothelial growth factor for neovascular agerelated macular degeneration. Cochrane Database Syst. Rev..

[5] Martin D.F., Maguire M.G., Fine S.L., Ying G.S., Jaffe G.J., Grunwald J.E., Toth C., Redford M., Ferris F.L., Comparison of Age-related Macular Degeneration Treatments Trials (CATT) Research Group (2012). Ranibizumab and bevacizumab for treatment of neovascular age-related macular degeneration: Two-year results. Ophthalmology.

[6] Brown D.M., Michels M., Kaiser P.K., Heier J.S., Sy J.P., Ianchulev T. (2009). Ranibizumab versus verteporfin photodynamic therapy for neovascular age-related macular degeneration: Two-year results of the ANCHOR study. Ophthalmology.

[7] Heier J.S., Brown D.M., Chong V., Korobelnik J.F., Kaiser P.K., Nguyen Q.D., Kirchhof B., Ho A., Ogura Y., Yancopoulos G.D. (2012). Intravitreal aflibercept (VEGF trap-eye) in wet age-related macular degeneration. Ophthalmology.

[8] Abu Dail Y., Seitz B., Sideroudi H., Abdin A.D. (2023). Impact of intravitreal ranibizumab, aflibercept and bevacizumab on retinal ganglion cell and nerve fibre layer thickness in Neovascular age-related macular degeneration. Acta Ophthalmol..

[9] Makri O.E., Vavvas D., Plotas P., Pallikari A., Georgakopoulos C.D. (2017). The effect of ranibizumab on normal neurosensory retina in the eyes of patients with exudative age related macular degeneration. Open Ophthalmol. J..

[10] Beck M., Munk M.R., Ebneter A., Wolf S., Zinkernagel M.S. (2016). Retinal ganglion cell layer change in patients treated with anti-vascular endothelial growth factor for neovascular age-related macular degeneration. Am. J. Ophthalmol..

[11] Lee S.W., Sim H.E., Park J.Y., Kim J.S., Chang I.B., Park Y.S., Hwang J.H. (2020). Changes in inner retinal layer thickness in patients with exudative age-related macular degeneration during treatment with anti-vascular endothelial growth factor. Medicine.

[12] Kim S.Y., Yoon M.H., Chin H.S. (2020). Changes in the ganglion cell-inner plexiform layer after consecutive intravitreal injections of anti-vascular endothelial growth factor in age-related macular degeneration patients. Korean J. Ophthalmol..

[13] Inan Ü.Ü., Baysal Z., Inan S. (2019). Long-term changes in retinal layers in patients undergoing intravitreal ranibizumab for neovascular age-related macular degeneration: Retinal layers after anti-VEGF therapy. Int. Ophthalmol..

[14] Aşikgarip N., Temel E., Örnek K. (2021). Macular ganglion cell complex changes in eyes treated with aflibercept for neovascular age-related macular degeneration. Photodiagnosis Photodyn. Ther..

[15] Demir N., Sevincli S., Kayhan B., Sonmez M. (2021). Anatomical effects of intravitreal anti-vascular endothelial growth factor injections on inner layers of the lesion-free retina. Cutan. Ocul. Toxicol..

[16] Abdolrahimzadeh S., Gharbiya M., Formisano M., Bertini F., Cerini A., Pacella E. (2019). Anti-vascular endothelial growth factor intravitreal therapy and macular ganglion cell layer thickness in patients with neovascular age-related macular degeneration. Curr. Eye Res..

[17] Kim S.W., Woo J.E., Yoon Y.S., Lee S., Woo J.M., Min J.K. (2019). Retinal and Choroidal Changes after Anti Vascular Endothelial Growth Factor Therapy for Neovascular Age-related Macular Degeneration. Curr. Pharm. Des..

[18] de Vries V.A., Bassil F.L., Ramdas W.D. (2020). The effects of intravitreal injections on intraocular pressure and retinal nerve fiber layer: A systematic review and meta-analysis. Sci. Rep..

[19] Shin H.J., Kim S.N., Chung H., Kim T.E., Kim H.C. (2016). Intravitreal anti-vascular endothelial growth factor therapy and retinal nerve fiber layer loss in eyes with age-related macular degeneration: A meta-analysis. Investig. Ophthalmol. Vis. Sci..

[20] Jun S.Y., Hwang D.D.J. (2023). Short-term effect of intravitreal brolucizumab injections in patients with neovascular age-related macular degeneration on retinal nerve fiber layer thickness. Sci. Rep..

[21] Aljundi W., Daas L., Suffo S., Seitz B., Abdin A.D. (2024). First-year real-life experience with intravitreal faricimab for refractory neovascular age-related macular degeneration. Pharmaceutics.

[22] Abdin A.D., Aljundi W., El Jawhari K., Suffo S., Weinstein I., Seitz B. (2022). First year real life experience with intravitreal brolucizumab for treatment of refractory neovascular age-related macular degeneration. Front. Pharmacol..

[23] Abdin A.D., Suffo S., Bischoff-Jung M., Daas L., Pattmöller M., Seitz B. (2020). Advantages of a designated IVI center for a German university eye hospital. Der Ophthalmol..

[24] Huang Y., Gangaputra S., Lee K.E., Narkar A.R., Klein R., Klein B.E.K., Meuer S.M., Danis R.P. (2012). Signal quality assessment of retinal optical coherence tomography images. Investig. Ophthalmol. Vis. Sci..

[25] de Azevedo A.G.B., Takitani G.E.d.S., Godoy B.R., Marianelli B.F., Saraiva V., Tavares I.M., Roisman L. (2020). Impact of manual correction over automated segmentation of spectral domain optical coherence tomography. Int. J. Retin. Vitr..

[26] Lee Y.J., Yoo Y.J., Han S.B. (2020). Age-related changes of macular ganglion cell-inner plexiform layer thickness in Korean elderly subjects. Korean J. Ophthalmol..

[27] Zhang X., Francis B.A., Dastiridou A., Chopra V., Tan O., Varma R., Greenfield D.S., Schuman J.S., Huang D., Advanced Imaging for Glaucoma Study Group (2016). Longitudinal and cross-sectional analyses of age effects on retinal nerve fiber layer and ganglion cell complex thickness by fourier-domain OCT. Transl. Vis. Sci. Technol..

[28] Cui Q.N., Gray I.N., Yu Y., VanderBeek B.L. (2019). Repeated intravitreal injections of antivascular endothelial growth factors and risk of intraocular pressure medication use. Graefe’s Arch. Clin. Exp. Ophthalmol..

[29] Levin A.M., Chaya C.J., Kahook M.Y., Wirostko B.M. (2021). Intraocular pressure elevation following intravitreal anti-VEGF injections: Short- and long-term considerations. J. Glaucoma.

[30] Horsley M.B., Mandava N., Maycotte M.A., Kahook M.Y. (2010). Retinal nerve fiber layer thickness in patients receiving chronic anti-vascular endothelial growth factor therapy. Am. J. Ophthalmol..

[31] Martinez-de-la-Casa J.M., Ruiz-Calvo A., Saenz-Frances F., Reche-Frutos J., Calvo-Gonzalez C., Donate-Lopez J., Garcia-Feijoo J. (2012). Retinal nerve fiber layer thickness changes in patients with age-related macular degeneration treated with intravitreal ranibizumab. Investig. Ophthalmol. Vis. Sci..

[32] Sobacı G., Güngör R., Ozge G. (2013). Effects of multiple intravitreal anti-VEGF injections on retinal nerve fiber layer and intraocular pressure: A comparative clinical study. Int. J. Ophthalmol..

[33] Shin H.J., Shin K.C., Chung H., Kim H.C. (2014). Change of retinal nerve fiber layer thickness in various retinal diseases treated with multiple intravitreal antivascular endothelial growth factor. Investig. Ophthalmol. Vis. Sci..

[34] Parlak M., Oner F.H., Saatci A.O. (2015). The long-term effect of intravitreal ranibizumab on retinal nerve fiber layer thickness in exudative age-related macular degeneration. Int. Ophthalmol..

[35] Demirel S., Batioğlu F., Özmert E., Erenler F. (2015). The effect of multiple injections of ranibizumab on retinal nerve fiber layer thickness in patients with age-related macular degeneration. Curr. Eye Res..

[36] Jo Y.J., Kim W.J., Shin I.H., Kim J.Y. (2016). Longitudinal changes in retinal nerve fiber layer thickness after intravitreal anti-vascular endothelial growth factor therapy. Korean J. Ophthalmol..

[37] Sengul E.A., Artunay O., Kumral E.T., Yenerel M., Rasier R., Kockar A., Yuzbasioglu E. (2016). Retinal nerve fiber layer thickness changes in age-related macular degeneration treated with multiple intravitreal ranibizumab. J. Ocul. Pharmacol. Ther..

[38] Valverde-Megías A., Ruiz-Calvo A., Murciano-Cespedosa A., Hernández-Ruiz S., Martínez-de-la-Casa J.M., García-Feijoo J. (2019). Long-term effect of intravitreal ranibizumab therapy on retinal nerve fiber layer in eyes with exudative age-related macular degeneration. Graefe’s Arch. Clin. Exp. Ophthalmol..

[39] Hammel N., Belghith A., Weinreb R.N., Medeiros F.A., Mendoza N., Zangwill L.M. (2017). Comparing the rates of retinal nerve fiber layer and ganglion cell-inner plexiform layer loss in healthy eyes and in glaucoma eyes. Am. J. Ophthalmol..

[40] Hougaard J.L., Ostenfeld C., Heijl A., Bengtsson B. (2006). Modelling the normal retinal nerve fibre layer thickness as measured by Stratus optical coherence tomography. Graefe’s Arch. Clin. Exp. Ophthalmol..

[41] Bendschneider D., Tornow R.P., Horn F.K., Laemmer R., Roessler C.W., Juenemann A.G., Kruse F.E., Mardin C.Y. (2010). Retinal nerve fiber layer thickness in normals measured by spectral domain OCT. J. Glaucoma.

[42] Parikh R.S., Parikh S.R., Sekhar G.C., Prabakaran S., Babu J.G., Thomas R. (2007). Normal age-related decay of retinal nerve fiber layer thickness. Ophthalmology.

[43] Entezari M., Ramezani A., Yaseri M. (2014). Changes in Retinal Nerve Fiber Layer Thickness after Two Intravitreal Bevacizumab Injections for Wet Type Age-related Macular Degeneration. J. Ophthalmic Vis. Res..

[44] Wang L., Swaminathan S.S., Yang J., Barikian A., Shi Y., Shen M., Jiang X., Feuer W., Gregori G., Rosenfeld P.J. (2021). Dose-response relationship between intravitreal injections and retinal nerve fiber layer thinning in age-related macular degeneration. Ophthalmol. Retin..

[45] Waldstein S.M., Simader C., Staurenghi G., Chong N.V., Mitchell P., Jaffe G.J., Lu C., Katz T.A., Schmidt-Erfurth U. (2016). Morphology and visual acuity in aflibercept and ranibizumab therapy for neovascular age-related macular degeneration in the VIEW trials. Ophthalmology.

[46] Ahn J., Jang K., Sohn J., Park J.I., Hwang D.D.J. (2021). Effect of intravitreal ranibizumab and aflibercept injections on retinal nerve fiber layer thickness. Sci. Rep..

